# Case report: Concurrent intrathecal and intravenous pembrolizumab for metastatic melanoma with leptomeningeal disease

**DOI:** 10.3389/fonc.2024.1344829

**Published:** 2024-04-11

**Authors:** Xiang Dan, Mengxi Huang, Zhaochen Sun, Xiaoyuan Chu, Xin Shi, Yitian Chen

**Affiliations:** ^1^ Affiliated Jinling Hospital, School of Medicine, Nanjing University, Nanjing, China; ^2^ Department of Medical Oncology, Affiliated Jinling Hospital, School of Medicine, Nanjing University, Nanjing, China; ^3^ Department of Radiation Oncology, Affiliated Jinling Hospital, School of Medicine, Nanjing University, Nanjing, China; ^4^ Department of Orthopedics, Jinling Hospital, Nanjing, China

**Keywords:** leptomeningeal disease, metastatic melanoma, pembrolizumab, cancer, immunotherapy

## Abstract

Leptomeningeal disease (LMD) is a serious cancer complication associated with poor prognosis. Approximately 5%–25% of patients with melanoma develop LMD. Currently, no standard treatment protocol exists and very few cases have been reported. Despite ongoing advances in new therapies, treatment options for LMD remain limited. Herein, we report a case of intrathecal pembrolizumab administration in a patient with melanoma and LMD. Intrathecal pembrolizumab administration was feasible and safe at the doses tested. Drawing from this case, along with our expertise and the existing evidence on systemic immunotherapy, we propose that an immunotherapy approach involving intrathecal administration for patients with LMD from melanoma warrants additional exploration in clinical trials.

## 1 Introduction

Leptomeningeal disease (LMD) entails malignant seeding to the connective tissue layers of soft meninges (arachnoid and pia mater). The prevalence of LMD in solid cancers is as follows: breast cancer (12%–35%), lung cancer (10%–26%), melanoma (5%–25%), and gastrointestinal malignancies (4%–14%) (1). Thakkar et al. (2) reported melanoma as having the highest incidence of LMD (23%), followed by lung and breast cancers. While novel treatment options have arisen for metastatic melanoma, such as targeted therapy and immunotherapy, patients with LMD related to melanoma have a poor prognosis, with an overall survival of approximately only 3.5 months (3, 4). In addition, patients with LMD have been excluded from most clinical trials; therefore, LMD remains relatively unexplored, with limited treatment approaches and few clinical trials.

Immunotherapy can double the overall survival rate of melanoma patients with brain metastases (5). However, data on the safety and efficacy of immunotherapy in patients with LMD are limited. To the best of our knowledge, this is the first report of intrathecal (IT) pembrolizumab administration in a patient with metastatic melanoma and LMD. This case report demonstrates the safety and feasibility of IT immunotherapy and supports further clinical studies in this challenging patient population.

## 2 Case description

**Figure 1:**
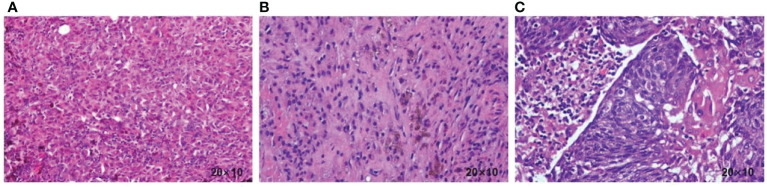
**(A)** Histopathology of the scalp tumor resection specimen showing a malignant melanoma. **(B)** Histopathology of the cervical lymph node resection specimen showing a malignant melanoma. **(C)** Histopathology of the cervical resection specimen, showing squamous cell carcinoma.

A 58-year-old female patient presented with persistent nonhealing and progressive enlargement of an ulcerated mass in the left temporoparietal region. She underwent radical scalp skin lesion excision under general anesthesia in September 2021 for continuous bleeding in the mass region. The postoperative pathology report (**Figure 1A**) clearly demonstrated cutaneous melanoma (approximately 2.4 cm × 2 cm) with Breslow thickness (1.1 cm) and ulceration (0.1 cm) from the closest point of the basal cut edge. Positron emission tomography/computed tomography (PET/CT) scans were performed, and it was observed that the melanoma had metastasized to the left cervical lymph node, and a metabolic mass was present in the cervix, which was considered a cervical cancer. In October 2021, the patient underwent radical cervical cancer surgery and left cervical lymph node dissection. Postoperative pathological examination showed metastatic melanoma in the left cervical lymph node (**Figure 1B**) and stage Ib cervical squamous cell carcinoma (7 cm × 5.5 cm × 3 cm) without lymph node metastasis (**Figure 1C**). Combined with the first postoperative pathology report, cutaneous melanoma was diagnosed as stage IIIB (T2bN1bM0). Scalp mass and blood genetic tests showed a BRAF V600E mutation, positive PD-L1 expression, and CPS of 10. The patient was then administered dabrafenib (150 mg, bid, PO) and trametinib (2 mg, qd, PO) for adjuvant therapy but stopped due to a grade 3 gastrointestinal reaction with fever (fever peak 41 °C) after 1 week. The patient was treated with pembrolizumab (200 mg, q3w, IV) between November 2021 and March 2023 as adjuvant therapy. In the subsequent follow-ups, no progression was observed in the cervical cancer primary site until the patient died.

**Figure 2:**
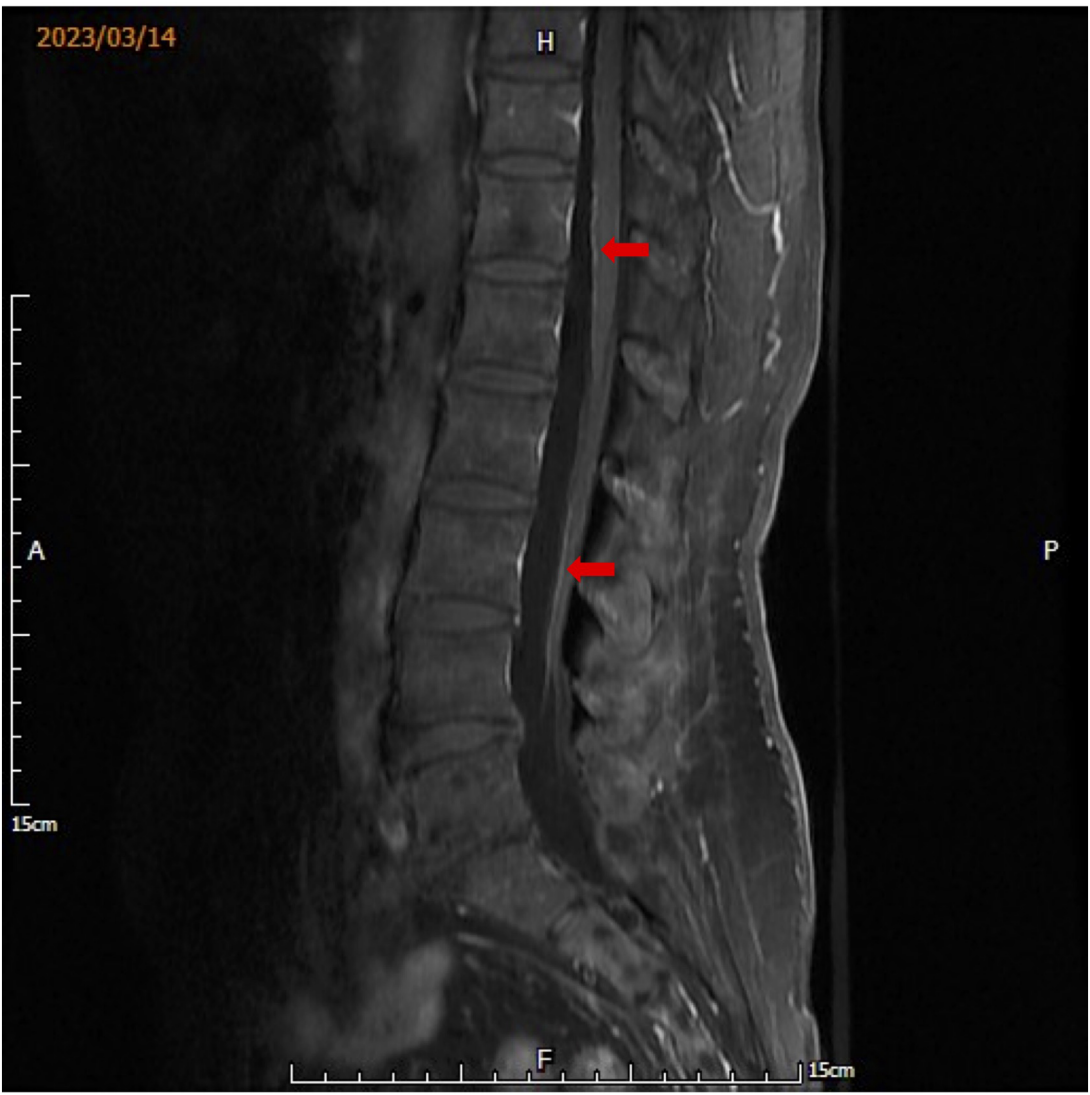
Whole spinal cord MRI showing multiple foci of striated enhancement of the spinal nerves, assessing metastasis in the soft spine (arrows are foci of spinal nerve enhancement).

In April 2022, cranial magnetic resonance imaging (MRI) revealed multiple brain metastases. Dabrafenib (75 mg, bid, PO) and trametinib (1 mg, qd, PO) both at half dose of adjuvant therapy, considering patient tolerance,were added to the treatment and kept at the same dosage in the later combination therapy. During subsequent follow-ups, cranial MRIs revealed disease stabilization until February 2023. The patient developed bilateral lower limb weakness, and whole spinal cord MRI showed leptomeningeal disease in March 2023 (**Figure 2**). Subsequently, whole-brain radiotherapy at a regimen of 30 Gy/10 F was performed, during which pembrolizumab combined with dabrafenib and trametinib therapy was resumed.

**Figure 3:**
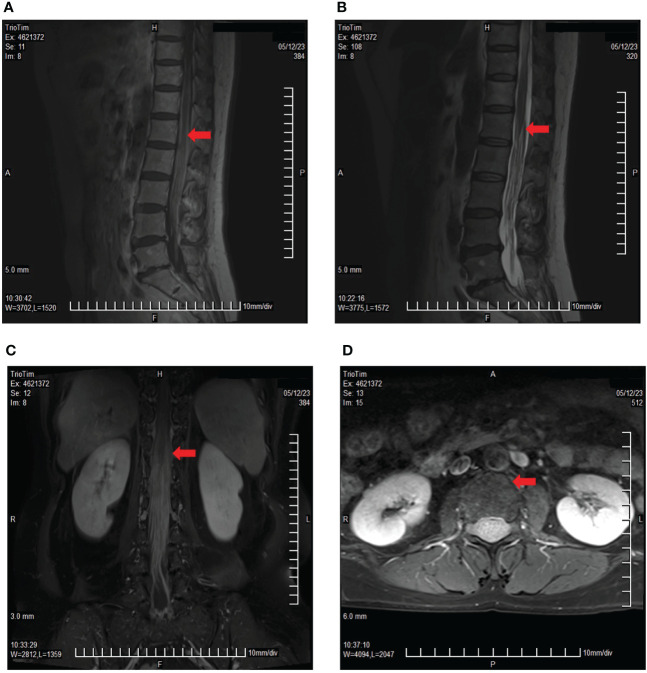
Lumbar spine MRI showing multiple cord shadows at the cauda equina, which were significantly thickened and increased, indicating the rapid progression of the disease. **(A)** Sagittal T1 sequence; **(B)** Sagittal T2 sequence; **(C)** Coronal enhancement sequence; **(D)** Transverse enhancement sequence (arrows indicate tumor foci).

**Figure 4:**
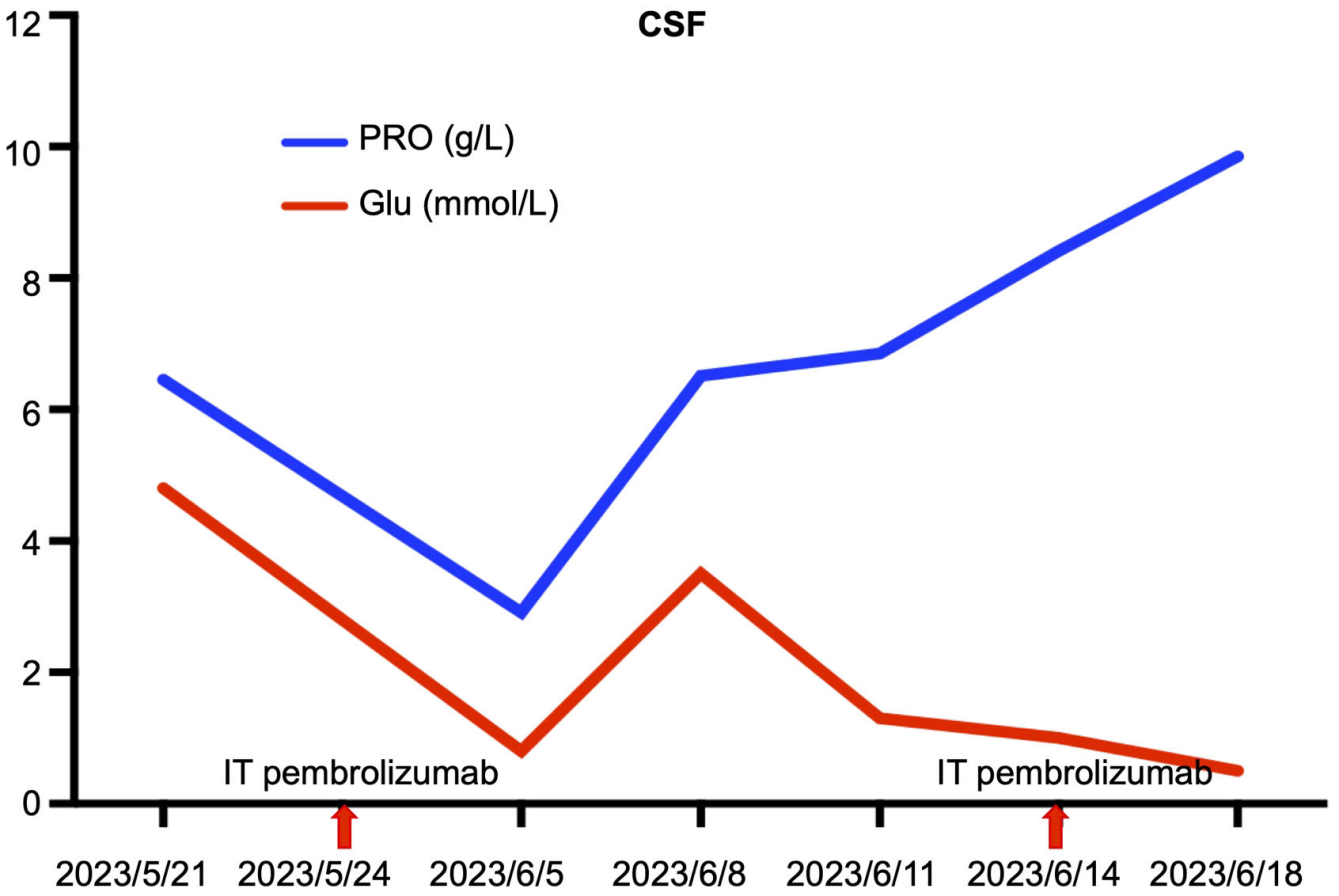
Albumin and glucose concentrations in CSF after IT pembrolizumab therapy.

Disease progression was evaluated 2 months later using MRI, and two cycles of cadonilimab combined with dabrafenib and trametinib were administered. In May 2023, the patient presented with paraplegia, subumbilical hyperalgesia (grade 1 muscle strength of both lower limbs), nausea, vomiting, and headache. Cranial MRI was used to assess disease progression (**Figure 3**). A reservoir was placed, and cerebrospinal fluid (CSF) cytopathology was negative. After MDT evaluation, we decided to adjust the regimen to IT injections of 25 mg pembrolizumab and 175 mg intravenous pembrolizumab (q3w), with continuation of dabrafenib and trametinib. No adverse events were reported following the first IT infusion. The symptoms of nausea, vomiting, and headache were relieved, and the muscle strength of both lower limbs improved to grade 2. Albumin and glucose levels in the CSF decreased (**Figure 4**). In June 2023, nausea, vomiting, and headache were aggravated, and after communication with the patient and her family, we treated her with mannitol and dexamethasone, and the symptoms improved. The patient underwent a second IT infusion of pembrolizumab (25 mg) and IV pembrolizumab (175 mg) 3 weeks after the first dose but exhibited no significant symptom relief.

**Figure 5:**
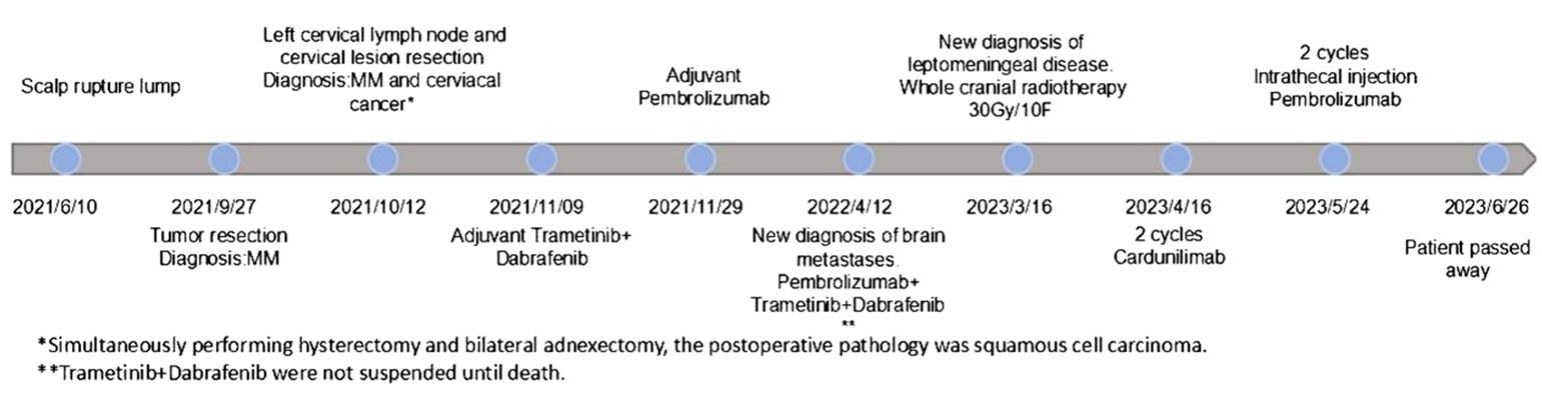
Clinical timeline.

The patient subsequently experienced disease progression with intermittent respiratory and cardiac arrest and died 2 weeks after the second IT pembrolizumab infusion from unrecoverable respiratory arrest (**Figure 5**). After IT administration, we did not assess the radiographic response due to deterioration of the patient’s clinical condition.

## 3 Discussion

To the best of our knowledge, this is the first case report of a metastatic melanoma patient with LMD treated with pembrolizumab IT. Two IT infusions were administered without any infusion reactions and no adverse events were directly associated with their administration. The symptoms were relieved after the first IT dose, and CSF analysis was performed. Due to the limitation of not assessing imaging, we cannot conclude that the patient benefited from IT therapy. However, this case showed the feasibility of IT pembrolizumab administration in melanoma patients with LMD, which may have the potential for extrapolation across LMD from other solid tumors and supports further studies to identify its safety and efficacy.

Radiation, systemic therapies, or a limited number of IT therapies are the current treatment options for patients with LMD (6, 7). As systemically administered agents find it difficult to enter the central nervous system (CNS) due to the blood–brain and blood–cerebrospinal fluid barriers (BBB and BCSF) (8, 9), direct IT infusion is an attractive treatment strategy. A pool analysis of 27 published studies with 2,161 patients (79 patients with melanoma) receiving IT therapy for LMD demonstrated that 44.7% of patients showed clinical improvement and 29.9% showed negative CSF cytology after treatment (10). The most commonly used drugs are chemotherapeutic agents with limited data on IT immunotherapy. The administration of interleukin-2 (IL-2) via the IT route in patients with metastatic melanoma or lung adenocarcinoma has been reported previously, demonstrating the feasibility of IT immunotherapy (11–13). A study retrospectively analyzed 43 patients with metastatic melanoma with LMD treated with IT IL-2 and demonstrated a survival benefit in a subset of patients; however, significant toxicity concerns were noted (14).

Currently, the approved immune checkpoint inhibitors (ICIs) for metastatic melanoma therapy are IgG monoclonal antibodies with neonatal Fc receptor (FcRn) binding, which can cross an intact BBB (15). FcRn enables antibodies to enter cells residing in the choroid plexus via endocytosis and to be released into the CSF. The outcomes of ICIs used in patients with LMD from different malignancies have been reported (16–19). One study enrolled 18 patients with solid tumors and LMD, and all patients received ipilimumab and nivolumab treatment. The 3-month survival rate was 44% (8/18), and the study met the primary endpoint (16). Furthermore, a single-arm, phase-2 clinical trial enrolled 20 patients with breast, lung, and ovarian cancers along with LMD manifestations (17). The patients received IV pembrolizumab (200 mg) every 3 weeks. The findings showed that 60% of the patients survived for 3 months after enrollment. In another phase II clinical trial, pembrolizumab monotherapy induced a CNS response in 38% of patients at week 12, and the response was defined as cytological, radiological, or clinical (18). Thus, pembrolizumab is a feasible and relatively safe treatment option for LMD. However, the ABC study enrolled four patients with LMD receiving intravenous nivolumab, and none of the patients achieved intracranial responses (19); therefore, better administration strategies need to be explored. Recently, studies on the use of IT ICIs for LMD (20–22) have shown this to be a promising approach for LMD treatment. A case report of IT administration of pembrolizumab in a refractory triple-negative breast cancer patient with LMD demonstrated the feasibility and safety of this treatment (20). To the best of our knowledge, this is the only reported case of pembrolizumab IT administration. As our patient was treated with adjuvant pembrolizumab for more than 1 year with good tolerance and our experience of using pembrolizumab in patients with metastatic melanoma (pembrolizumab has been approved in China for metastatic melanoma but nivolumab has no melanoma indication in China), we considered the IT pembrolizumab strategy. A phase I/Ib study of nivolumab IT in patients with LMD from metastatic melanoma also revealed the safety and good tolerance of this approach, as no unexpected toxicity was observed (22). The study enrolled 25 patients treated with IT nivolumab monotherapy in cycle 1, and IV nivolumab was administered in subsequent cycles. The IT escalation doses were 5, 10, 20, and 50 mg of nivolumab, and no dose-limiting toxicity was observed. The recommended IT dose of nivolumab is 50 mg (IV, 240 mg) administered every 2 weeks. This study provides guidance for dose selection in our patient, as pembrolizumab has the same molecular weight as nivolumab. Considering that the body surface area of the patient was large and that there were no reports on the use of IT pembrolizumab in metastatic melanoma with LMD, we administered an IT dose of 25 mg with 175 mg IV pembrolizumab. The administration schedule was chosen every 3 weeks for consistency with pembrolizumab systemic infusion. It was considered a conservatively safe schedule given the limited literature on the safety and kinetics of IT PD-1 inhibitors.

Despite our findings, we cannot conclude that the patient may have benefitted from the two doses of IT infusion. Nevertheless, IT pembrolizumab might have contributed to temporary symptom relief and albumin and glucose decline in the CSF. Recently, a real-world study of IT anti-PD-1 treatment in patients with metastatic melanoma and LMD was reported (23). The study enrolled seven patients receiving IT nivolumab (n = 4) or pembrolizumab (n = 3). Four patients responded to treatment with symptom improvement and reduction or disappearance of linear enhancement on MRI, while three patients developed progressive disease. With a median follow-up of 13.3 weeks, the median intracranial progression-free survival (IPFS) and median OS for IT anti-PD-1 were 16.1 and 20.3 weeks, respectively. All treatment-related adverse events were of grade 1–2. Based on our case and the current evidence, we posit that the IT immunotherapy treatment approach for patients with LMD from melanoma is well tolerated and effective and merits further investigation in clinical trials.

## 4 Conclusion

In this study, we demonstrated the feasibility and safety of intrathecal pembrolizumab at the tested dosage. We believe that our study makes a significant contribution to the literature because makes a valuable contribution to the existing literature by addressing the critical and underexplored issue of leptomeningeal disease (LMD) in melanoma patients. As there is currently no standard treatment protocol for LMD, the findings of this study open new possibilities for therapeutic interventions. The results emphasize the need for further research on intrathecal immunotherapy, offering a potential avenue for improving outcomes in patients with LMD, which is a significant contribution to the oncology literature.

## Data availability statement

The original contributions presented in the study are included in the article/supplementary material. Further inquiries can be directed to the corresponding authors.

## Ethics statement

Written informed consent was obtained from the individual(s) for the publication of any potentially identifiable images or data included in this article.

## Author contributions

XD: Conceptualization, Data curation, Investigation, Writing – original draft, Writing – review & editing. MH: Conceptualization, Data curation, Investigation, Writing – original draft. ZS: Conceptualization, Investigation, Writing – review & editing. XC: Conceptualization, Formal analysis, Project administration, Supervision, Writing – original draft. XS: Conceptualization, Data curation, Formal analysis, Project administration, Resources, Writing – original draft. YC: Conceptualization, Funding acquisition, Investigation, Methodology, Resources, Supervision, Visualization, Writing – original draft.

## References

[1] Ferguson SD Bindal S Bassett RL Haydu LE McCutcheon IE Heimberger AB . Predictors of Treatment of Leptomeningeal Metastases (Carcinomatous Meningitis). SD; Bindal survival in metastatic melanoma patients with leptomeningeal disease (LMD). J Neurooncol. (2019) 142:499–509. doi: 10.1007/s11060-019-03121-2. Alexis Demopoulos M, Brown P, M.: Ferguson Publishing Company (2018). 30847840

[2] Thakkar JP Kumthekar P Dixit KS Stupp R Lukas RV . Leptomeningeal metastasis from solid tumors. Neurol Sci. (2020) 411:116706. doi: 10.1016/j.jns.2020.116706 32007755

[3] Ferguson SD Bindal S Bassett RL Haydu LE McCutcheon IE Heimberger AB . Predictors of survival in metastatic melanoma patients with leptomeningeal disease (LMD). J Neurooncol. (2019) 142:499–509. doi: 10.1007/s11060-019-03121-2 30847840

[4] Chorti E Kebir S Ahmed MS Keyvani K Umutlu L Kanaki T . Leptomeningeal disease from melanoma-poor prognosis despite new therapeutic modalities. Eur J Cancer. (2021) 148:395–404. doi: 10.1016/j.ejca.2021.02.016 33789203

[5] Iorgulescu JB Harary M Zogg CK Ligon KL Reardon DA Hodi FS . Improved risk-adjusted survival for melanoma brain metastases in the era of checkpoint blockade immunotherapies: results from a national cohort. Cancer Immunol Res. (2018) 6:1039–45. doi: 10.1158/2326-6066.CIR-18-0067 PMC6230261 30002157

[6] Glitza IC Smalley KSM Brastianos PK Davies MA McCutcheon I Liu JKC . Leptomeningeal disease in melanoma patients:an update to treatment, challenges, and future directions. Pigment Cell Melanoma Res. (2020) 33:527–41. doi: 10.1111/pcmr.12861 PMC10126834 31916400

[7] Le Rhun E Weller M Brandsma D Van den Bent M de Azambuja E Henriksson R . EANO–ESMO Clinical Practice Guidelines for diagnosis, treatment and follow-up of patients with leptomeningeal metastasis from solid tumours. Ann Oncol. (2017) 28:iv84–99. doi: 10.1093/annonc/mdx221 28881917

[8] Boire A Brastianos PK Garzia L Valiente M . Brain metastasis. Nat Rev Cancer. (2020) 20:4–11. doi: 10.1038/s41568-019-0220-y 31780784

[9] Arvanitis CD Ferraro GB Jain RK . The blood-brain barrier and blood-tumour barrier in brain tumours and metastases. Nat Rev Cancer. (2020) 20:26–41. doi: 10.1038/s41568-019-0205-x 31601988 PMC8246629

[10] Palmisciano P Watanabe G Conching A Ogasawara C Vojnic M d’amico RS . Intrathecal therapy for the management of leptomeningeal metastatic disease: a scoping review of the current literature and ongoing clinical trials. J Neurooncol. (2022) 160(1):79–100. doi: 10.1007/s11060-022-04118-0 35999434

[11] Heimans JJ Wagstaff J Schreuder WO Wolbers JG Baars JW Polman CH . Treatment of leptomeningeal carcinomatosis with continuous intraventricular infusion of recombinant interleukin-2. Surg Neurol. (1991) 35:244–7. doi: 10.1016/0090-3019(91)90079-O 1996455

[12] List J Moser RP Steuer M Loudon WG Blacklock JB Grimm EA . Cytokine responses to intraventricular injection of interleukin 2 into patients with leptomeningeal carcinomatosis: rapid induction of tumor necrosis factor alpha, inter-leukin 1 beta, interleukin 6, gamma-interferon, and soluble interleukin 2 receptor (Mr 55,000 Protein). Cancer Res. (1992) 52:1123–8. 1737371

[13] Fathallah-Shaykh HM Zimmerman C Morgan H Rushing E Schold SC Jr Unwin DH . Response of primary leptomeningeal melanoma to intrathecal recombinant interleukin-2. A Case Rep Cancer. (1996) 77:1544–50. doi: 10.1002/(SICI)1097-0142(19960415)77:8<1544::AID-CNCR18>3.0.CO;2-# 8608541

[14] Glitza IC Rohlfs M Guha-Thakurta N Bassett RL Bernatchez C Diab A . Retrospective review of metastatic melanoma patients with leptomeningeal disease treated with intrathecal interleukin-2. ESMO Open. (2018) 3:e000283. doi: 10.1136/esmoopen-2017-000283 29387478 PMC5786950

[15] Van Bussel MTJ Beijnen JH Brandsma D . Intracranial antitumor responses of nivolumab and ipilimumab: a pharmacodynamic and pharmacokinetic perspective, a scoping systematic review. BMC Cancer. (2019) 19:519. doi: 10.1186/s12885-019-5741-y 31146733 PMC6543612

[16] Brastianos PK Strickland MR Lee EQ Wang N Cohen JV Chukwueke U . Phase II study of ipilimumab and nivolumab in leptomeningeal carcinomatosis. Nat Commun. (2021) 12:5954. doi: 10.1038/s41467-021-25859-y 34642329 PMC8511104

[17] Brastianos PK Lee EQ Cohen JV Tolaney SM Lin NU Wang N . Single-arm, open-label Phase 2 trial of pembrolizumab in patients with leptomeningeal carcinomatosis. Nat Med. (2020) 26:1280–4. doi: 10.1038/s41591-020-0918-0 32483359

[18] Naidoo J Schreck KC Fu W Hu C Carvajal-Gonzalez A Connolly RM . Pembrolizumab for patients with leptomeningeal metastasis from solid tumors: efficacy, safety, and cerebrospinal fluid biomarkers. J Immunother Cancer. (2021) 9:e002473. doi: 10.1136/jitc-2021-002473 34380662 PMC8359453

[19] Long GV Atkinson V Lo S Sandhu S Guminski AD Brown MP . Combination nivolumab and ipilimumab or nivolumab alone in melanoma brain metastases: a multicentre randomised phase 2 study. Lancet Oncol. (2018) 19:672–81. doi: 10.1016/S1470-2045(18)30139-6 29602646

[20] Fortin Ensign SP Yancey E Anderson KS Mrugala MM . Safety and feasibility of intrathecal pembrolizumab infusion in refractory triple negative breast cancer with leptomeningeal disease: A case report. Curr problems cancer. Case Rep. (2021) 4:100103. doi: 10.1016/j.cpccr.2021.100103

[21] Aquilanti E Brastianos PK . Immune checkpoint inhibitors for brain metastases: a primer for neurosurgeons. Neurosurgery. (2020) 87:E281–8. doi: 10.1093/neuros/nyaa095 PMC7426188 32302389

[22] Glitza Oliva IC Ferguson SD Bassett R Foster AP John I Hennegan TD . Concurrent intrathecal and intravenous nivolumab in leptomeningeal disease: phase 1 trial interim results. Nat Med. (2023) 29:898–905. doi: 10.1038/s41591-022-02170-x 36997799 PMC10115650

[23] Zhen J Zhang X Cai L Chen L Lai M Li D . Intrathecal anti-PD-1 treatment in patients with metastatic melanoma (MM) with leptomeningeal disease (LMD): Real-world data and evidence. J Clin Oncol. (2023) 41:e21516–6. doi: 10.1200/JCO.2023.41.16_suppl.e21516 39422814

